# Levothyroxine Timing during Ramadan: A Randomized Clinical Trial

**DOI:** 10.1155/2023/2565031

**Published:** 2023-02-10

**Authors:** Moeber Mahzari, Fahad Al Remthi, Ibrahim Ajwah, Mohammed Al Hazmi, Wesam Moafa, Awad Al Shahrani, Sameerah Al Shehri, Motasim Badri

**Affiliations:** ^1^College of Medicine, King Saud Bin Abdulaziz University for Health Sciences, Riyadh, Saudi Arabia; ^2^King Abdullah International Medical Research Center, Riyadh, Saudi Arabia; ^3^Department of Medicine, Ministry of the National Guard-Health Affairs, Riyadh, Saudi Arabia; ^4^Diabetes and Endocrine Center, King Abdullah Hospital, Ministry of Health, Bisha, Saudi Arabia; ^5^Department of Epidemiology and Biostatistics, College of Public Health and Health Informatics, King Saud Bin Abdulaziz University for Health Sciences, Riyadh, Saudi Arabia

## Abstract

**Introduction:**

Hypothyroidism requires lifelong thyroid hormone replacement with levothyroxine. For most hypothyroid patients fasting during Ramadan, compliance with the administration procedure is a challenge. This study aimed to determine the impact of different administration times of levothyroxine on thyroid-stimulating hormone (TSH) and free T4 (FT4) levels before and after the holy month of Ramadan. *Materials and Methodology*. Hypothyroid patients taking levothyroxine were randomized to 3 groups during Ramadan: group 1, 30 minutes before the iftar meal; group 2, 3-4 hours after the iftar meal, with no food taken for at least 1 hour after the meal; group 3, they were not given specific instructions for taking levothyroxine during Ramadan. Thyroid function tests were performed within 2 weeks before Ramadan and within 2 weeks after Ramadan. Pre- and post-Ramadan TSH and free T4 levels were compared. Mixed-effects analyzes were performed to identify factors associated with changes in TSH and FT4 levels.

**Results:**

Compliance was lower in patients taking levothyroxine 3-4 hours after iftar. In addition, the majority of patients who had not received a specific recommendation took levothyroxine 30 minutes before iftar. There was a statistically significant increase in TSH (*P*=0.006) and FT4 (*P*=0.044) levels after Ramadan. In multivariate analysis, the cause of hypothyroidism (Hashimoto's; postthyroidectomy; compared to postradioactive iodine) and levothyroxine dose significantly affected FT4 levels. In contrast, no variable was significantly associated with TSH level. The timing of levothyroxine intake during Ramadan did not significantly affect TSH or FT4 levels.

**Conclusion:**

TSH and FT4 significantly increased after Ramadan. However, the timing of levothyroxine intake per se had no influence on TSH or free T4 levels. Therefore, hypothyroid patients might take levothyroxine either 30 minutes or 3-4 hours after iftar with no meal for 1 hour, depending on preference.

## 1. Introduction

Hypothyroidism is a form of thyroid gland disorder, usually due to iodine deficiency, autoimmune thyroid disease in iodine-replete countries, postthyroidectomy, or radioactive iodine therapy. Hypothyroidism results in numerous symptoms and metabolic changes due to decreased production of thyroid hormones and consequently decreased function of the target tissue [1–4].

The prevalence of overt hypothyroidism, defined as an elevated level of thyroid-stimulating hormone (TSH) above the normal reference range along with reduced thyroxine (T4) level, is estimated to be 1-2%, rising to 7% in the elderly population [2, 5].

The Saudi population has a high prevalence of hypothyroidism. In a retrospective study conducted in a primary healthcare center, 29.1% (1125 of 3872 patients) were found to have hypothyroidism. In addition, a cross-sectional study in the northern region of Saudi Arabia found a prevalence of 25.5% [2, 6].

Fasting during the month of Ramadan is an Islamic obligation and a great worship. Muslims are obliged to refrain from food and drink between dawn and dusk. For patients with chronic diseases such as hypothyroidism, it is necessary to adjust the timing of taking medication to the new eating and sleeping habits [7].

Levothyroxine is the mainstay of treatment for hypothyroidism. Levothyroxine tablets achieve up to 80% bioavailability when taken on an empty stomach. However, if taken near food, the amount of the drug absorbed may become less [8, 9].

To maximize the absorption of levothyroxine, the American Thyroid Association (ATA) and other societies recommend taking levothyroxine 30–60 minutes before breakfast or before bedtime with at least a 3-hour interval from the evening meal [10, 11].

Although administration of levothyroxine during nonfasting periods significantly reduces absorption of levothyroxine, many patients find it challenging to adhere to this recommendation. According to McMillan et al., up to 20% of patients with hypothyroidism have taken levothyroxine with a meal [9]. During Ramadan, this is even more challenging.

The meal plan during Ramadan consists of 2 main meals. The predawn meal before the fast begins (Suhoor) and the iftar with a possible third meal in the evening in between. To ensure adequate food intake before dawn during Ramadan, many Muslims sleep less at night. This significant change in dietary habits and sleep cycle is an obstacle that prevents most hypothyroid patients from adhering to levothyroxine recommendations [7].

There are many studies from different regions of the world investigating the timing of levothyroxine intake during Ramadan. Most of these studies are either retrospective or prospective cohort studies, with a few studies from Saudi Arabia and the Middle Eastern regions. This randomized controlled trial aimed to evaluate two-time points for taking levothyroxine during Ramadan.

## 2. Materials and Methodology

### 2.1. Participants

The study participants were patients treated in adult endocrinology clinics in King Abdulaziz Medical City-Riyadh (KAMC-R). Study participants included adult patients diagnosed with primary hypothyroidism of any cause, who had been on treatment for at least 6 months, and who planned to fast during Ramadan. Exclusion criteria included pregnancy, multiple comorbid conditions, and active malignancy. Ethical approval was granted by the Institutional Review Board of King Abdullah International Medical Research Center with approval number 0263/22 dated February 9, 2022. Informed consent was obtained from all participants.

### 2.2. Sample Size

The calculated sample size was 24 patients in each group based on a TSH standard deviation of 1.5 (uIU/mL) to find a difference of 1.0 (uIU/mL) in TSH level between the groups at a 95% confidence level and 80% power.

### 2.3. Study Design and Protocol

Our study was conducted from March to July 2022 using a randomized controlled design. Participants were divided into three groups using block randomization.

  Group 1: took levothyroxine 30 minutes before iftar meal.
  Group 2: took levothyroxine 3-4 hours after the iftar meal, with no food taken for at least 1-hour after.
  Group 3: received no specific advice on taking levothyroxine during Ramadan.

All patients were instructed to document the timing of daily levothyroxine intake, and these documents were collected and reviewed by the research team to assess the compliance rate. TSH levels and free T4 (FT4) levels were obtained in all participants within 2 weeks before and after Ramadan.

Baseline characteristics including sex, age, weight, levothyroxine dosage, cause of hypothyroidism, medications affecting levothyroxine absorption, as well as TSH and FT4 levels were collected for all participants. All laboratory tests were done in the same laboratory with the same procedure and equipment.

### 2.4. Statistical Analysis

Continuous data were summarized as means (SD), medians (interquartile range (IQR)), and ranges (minimum and maximum); categorical data were summarized as frequencies (%). Categorical data were compared with the *χ*^2^ test and continuous data with the Wilcoxon test or the Friedman test. Mixed-effects analyses were performed to identify factors associated with changes in TSH and FT4 levels. All patient demographic and clinical characteristics were considered for analysis. Factors that were found to be significant in the univariate analysis were included in the final multivariate model. All tests were two-sided and a *P* value <0.05 was considered significant. IBMSPSS statistical software (version 22, IBMSPSS Corp, OK, USA) was used to perform the analysis.

## 3. Results

**Figure 1:**
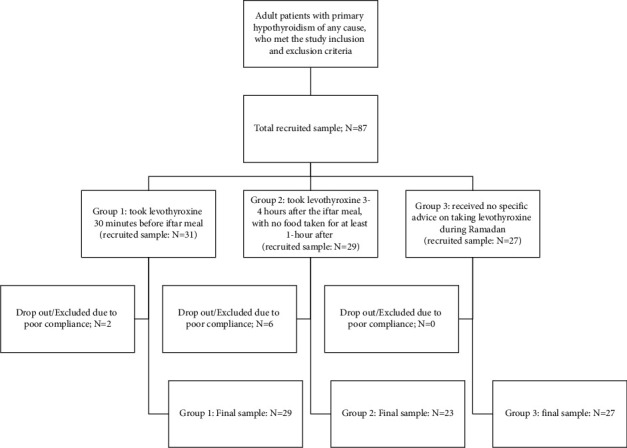
Participants flow diagram.

The study enrolled 87 patients, of whom 31 were assigned to group 1, 29 to group 2, and 27 to group 3. Eight participants were excluded from the final analysis because of inadequate compliance with levothyroxine (compliance of less than 50%). Six of the eight excluded participants were from group 2 and two were from group 1 (Figure 1).

Baseline characteristics of all patients participating in the study are shown in Table 1. A total of 29 patients were in group 1 (taking levothyroxine 30 minutes before iftar), 23 patients were in group 2 (taking levothyroxine 4 hours after iftar), and 27 patients were in group 3 (no specific advice). 21 of the 27 patients in group 3 took levothyroxine before iftar and 6 patients took it about 4 hours after iftar.

**Figure 2:**
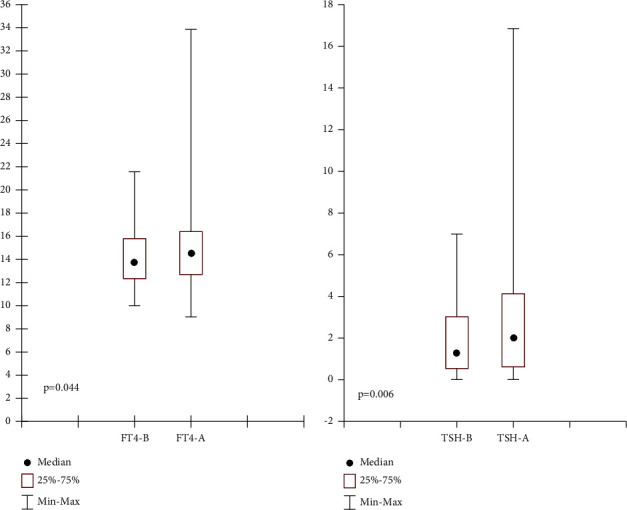
Wilcoxon pairwise test for overall FT4 and TSH values before (B) and after (A) Ramadan.

**Figure 3:**
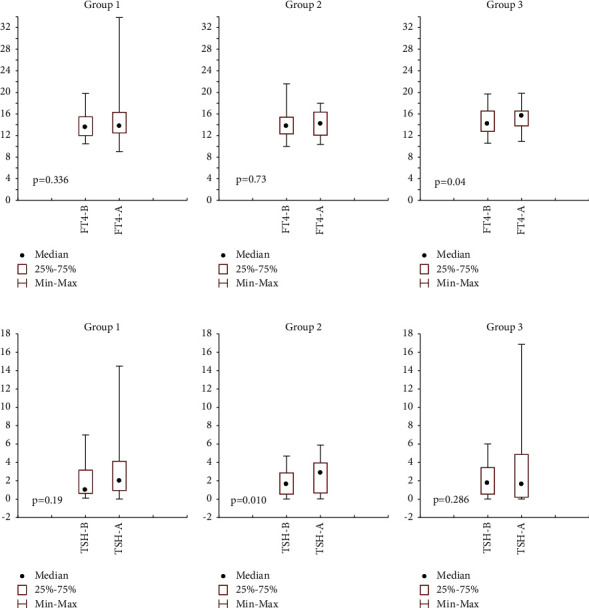
Wilcoxon pairwise test for FT4 and TSH values before (B) and after (A) Ramadan, stratified by the levothyroxine timing group (group 1: took levothyroxine 30 minutes before iftar; group 2: took levothyroxine 4 hours after iftar; group 3: no specific advice).

There were no statistically significant differences between the 3 groups in baseline variables such as age, sex, cause of hypothyroidism, duration of hypothyroidism, and TSH and FT4 levels before Ramadan. However, compliance with thyroxine was statistically significantly lower in patients taking levothyroxine 4 hours after iftar (*P*=0.012), as given in Table 2. There was a statistically significant increase in TSH (*P*=0.006) and FT4 (*P*=0.044) levels after Ramadan in the whole sample, as shown in Figure 2. Furthermore, in a further stratified analysis, the only significant difference for FT4 was observed in group 3 (*P*=0.04) and for TSH was observed in group 2 (*P*=0.010), as shown in Figure 3. However, in the mixed-effects multivariate analysis performed to identify factors associated with the changes in TSH and FT4 levels, the cause of hypothyroidism (Hashimoto's; *β* = 2.92, 95% CI: 1.00–5.15, and *P*=0.011, postthyroidectomy; *β* = 3.72, 95% CI: 1.43–6.02, and *p*=0.002 compared to postradioactive iodine), and levothyroxine dose (*β* = 0.03, 95% CI: 0.01–0.41, and *P* < 0.0001) significantly affected FT4 levels. In contrast, no variable was significantly associated with TSH levels. The timing of levothyroxine intake during Ramadan per se did not significantly affect TSH or FT4 levels (Table 3).

## 4. Discussion

This study was a randomized trial in patients with hypothyroidism to investigate the impact of levothyroxine timing during Ramadan fasting on TSH and FT4. The study also investigated the possible variables associated with the changes in TSH and FT4.

Changing the time of taking levothyroxine during Ramadan is challenging for both the patient and the physician, as the time for taking the drug is relatively limited and related to meals. Most physicians follow the recommendation of ATA/ETA when prescribing levothyroxine during Ramadan and recommend one of four times: 30 minutes before iftar, 3 hours after iftar, 30 minutes before Suhoor, or 3 hours after Suhoor, provided that the Suhoor meal was early enough [2, 10, 11].

Several retrospective studies and few prospective studies investigated the optimal timing for taking levothyroxine during Ramadan with conflicting results. In Saudi Arabia, very few studies have investigated the timing of levothyroxine intake during Ramadan.

Our study was performed in patients with different etiologies of hypothyroidism and reflects a wide spectrum of causes. TSH and FT4 increased significantly in the cohort after Ramadan. This finding suggests that compliance during Ramadan is challenging, even when reported as optimal by the patients. In our study, patient compliance was confirmed using a medication diary for the entire month. It ranged from 73% to 100% and yet both TSH and FT4 increased after Ramadan. Since both FT4 and TSH increased, it is possible that patients were less compliant with taking levothyroxine at the beginning of the month resulting in an increase in TSH after Ramadan. Then, toward the end of the month, intake became more precise, which was reflected in a higher FT4 than before Ramadan. Similar changes in thyroid function tests in the post-Ramadan period have been noted in other studies [12, 13]. Of note both studies measured TSH around 2 weeks after Ramadan, less than 6 weeks as reported by Koli et al. and 1-2 weeks after Ramadan as reported by Alsheikh et al.

On the contrary, El-Kaissi et al. in a randomized study, reported a significant decrease in plasma TSH levels when measured within 6 weeks after Ramadan in 50 patients taking levothyroxine 30 minutes before iftar, with a compliance rate of 69.4% [14]. In addition, a prospective study by Dabbous et al. showed that there was a significant decrease in serum T4 levels within 2 weeks after Ramadan [15]. In addition, a small prospective study in which a thyroid function test was performed 3 weeks after Ramadan showed no significant change in serum T4 levels [16].

In our study, we found that the lowest compliance rate was in group 2 (instructed to take levothyroxine 4 hours after iftar). Most patients who were excluded from the final analysis because of a compliance rate of less than 50% belonged to this group. This suggests that this time-point presents a greater challenge in terms of compliance. In addition, approximately 80% of patients who did not receive specific instructions on how to take levothyroxine choose to take it before iftar. This also suggests that patients prefer the timing before iftar. Regarding the optimal timing of levothyroxine intake, our study showed that the timing of levothyroxine intake had no significant effect on TSH levels or FT4 when adjusted for other variables in multivariate analysis. Even in patients who had not received specific instructions to take levothyroxine, changes in TSH and FT4 after Ramadan were similar. The lack of difference between the groups could be in fact a reflection that all patients took levothyroxine on a relatively empty stomach regardless of the medication time. In a recent randomized controlled trial, a significant increase in TSH levels 2 weeks after Ramadan compared with pre-Ramadan levels was observed in patients taking levothyroxine 3 hours after iftar or 1 hour before Suhoor [17]. However, it was not clear whether the comparison between the groups showed a difference.

Among the variables that influenced, the changes in FT4 and TSH after Ramadan may have been the cause of hypothyroidism. A diagnosis of Hashimoto's or thyroidectomy as the cause of hypothyroidism resulted in higher FT4 levels but no significant change in TSH levels compared with patients who had hypothyroidism after radioactive iodine ablation. Higher levothyroxine dose was also associated with higher FT4 levels after Ramadan. In contrast, no single variable alone was associated with the change in TSH level after Ramadan.

The strengths of our study include the relatively high compliance rate in all patients and the confirmation of compliance by keeping a medication diary for all patients. Moreover, group 3 who did not receive any specific instructions regarding thyroxine intake is a control group in the study. The fact that we had patients with different causes of hypothyroidism reflects the usual population of hypothyroidism. Limitations of the study include the small sample size and the fact that it was a single-center study. Of note, the timing of the thyroid function test being 2 weeks after Ramadan might have affected the study results. Future studies with TSH and FT4 measurement immediately after Ramadan with comparison to a thyroid function tests few weeks after Ramadan worth to be done.

## 5. Conclusion

Taking levothyroxine during Ramadan is challenging for patients. Before, iftar seems to be easier. Although TSH and FT4 are likely to change after Ramadan, the timing of levothyroxine intake was not a significant factor when all other variables were considered.

## Figures and Tables

**Table 1:** Demographic and clinical characteristics of the patients.

Factor	*n* (%)
Age	
Mean (SD)	50 (14.8)
Median (IQR)	50 (39–63)
Minimum-maximum	18–80
Gender	
Male	15 (19)
Female	64 (81)
Timing of thyroxine intake during Ramadan	
30 minutes before iftar	29 (36.7)
4 hours after iftar	23 (29.1)
No specific advice	27 (34.2)
Duration of hypothyroidism (years)	
Mean (SD)	6.4 (5.7)
Median (IQR)	4 (2–11)
Minimum-maximum	0.40–20
Average levothyroxine dose	
Mean (SD)	112.2 (38.8)
Median (IQR)	107 (92–143)
Minimum-maximum	25–200
FT4 level before Ramadan (reference range 9.0–19.0 pmol/L)	
Mean (SD)	14.1 (2.4)
Median (IQR)	13.7 (12.3–15.8)
Minimum-maximum	10–21.6
TSH level before Ramadan (reference range 0.35–4.94 mIU/L)	
Mean (SD)	1.9 (1.8)
Median (IQR)	1.3 (0.5–3)
Minimum-maximum	0.01–6.7
Cause of hypothyroidism	
Hashimoto	35 (44.3)
Postthyroidectomy	40 (50.6)
Postradioactive iodine	4 (5.1)
Other medications use	
PPI	7 (8.9)
Calcium	16 (20.3)
No medications	56 (70.9)

SD, standard deviation; IQR, interquartile range; PPI, proton pump inhibitors.

**Table 2:** Comparison of treatment-related characteristics by the treatment initiation group.

Characteristics	Treatment initiation time group			*P*
	30 minutes before iftar	4 hours after iftar	No specific advice	
Age	56 (40–65.5)	48 (39–66)	49 (37–55)	0.169
Gender, *n* (%)				0.953
Male	6 (20.7)	4 (17.4)	5 (18.5)	
Female	23 (79.3)	19 (82.6)	22 (81.5)	
Cause of hypothyroidism, *n* (%)				0.232
Hashimoto	15 (51.7)	11 (47.8)	9 (33.3)	
Postthyroidectomy	11 (37.9)	12 (52.2)	17 (63)	
Postradioactive iodine	3 (10.3)	(0)0	1 (3.7)	
Other medications use, *n* (%)				0.273
No medications	18 (62.1)	16 (69.6)	22 (81.5)	
PPI	4 (13.8)	3 (13)	0	
Calcium	7 (24.1)	4 (17.4)	5 (18.5)	
Duration of hypothyroidism (years)	3 (1–10.5)	5 (2–11)	4 (2–12)	0.311
FT4 level before Ramadan^*∗*^	13.6 (11.9–15.8)	13.7 (12.3–15.4)	14.23 (12.77 (16.6))	0.670
TSH level before Ramadan^*∗∗*^	0.97 (0.55–3.47)	1.63 (0.54–2.86)	1.7 (0.53–3.44)	0.942
Average levothyroxine dose	100 (87.5–125)	107 (75–150)	125 (100–150)	0.365
Compliance to levothyroxine	100 (100–100)	93 (73–100)	100 (93–100)	0.012

Unless otherwise stated; numbers are presented as median (interquartile range). PPI, proton pump inhibitors; ^*∗*^reference range: 9.0–19.0 pmol/L; ^*∗∗*^reference range: 0.35–4.94 mIU/L.

**Table 3:** Multivariate repeated-measuresmixed-effects models for factors associated with changes in free T4 and TSH.

Factor	FT4	*P*	TSH	*P*
	*β* (95% CI)		*β* (95% CI)	
Treatment initiation time				
30 minutes before iftar	0.48 (−0.68, 1.63)	0.414	−0.41 (−1.40, 0.58)	0.413
4 hours after iftar	−0.20 (−1.45, 1.06)	0.754	−0.62 (−1.70, 0.457)	0.254
No specific advice	1		1	
Cause of hypothyroidism				
Hashimoto	2.92 (1.00, 5.15)	0.011	−0.21 (−2.13, 1.07)	0.824
Postthyroidectomy	3.72 (1.43–6.02)	0.002	−1.91 (−3.89, 0.06)	0.058
Postradioactive iodine	1		1	
Other drug				
Calcium	0.08 (−1.16, 1.31)	0.903	−0.95 (−2.01, 0.11)	0.079
PPI	−0.48 (−2.18, 1.22)	0.577	−1.26 (−2.73, 0.20)	0.09
No medications	1		1	
Levothyroxin dose	0.03 (0.01, 0.41)	<0.0001	−0.002 (−0.01, 0.003)	0.157
Thyroxin compliance	0.18 (−0.03, 0.06)	0.414	−0.007 (−0.05, 0.03)	0.720

## Data Availability

The data used to support the findings of this study are available from the corresponding author upon request.
